# An Almond Meal-Rich Diet Improves Red Blood Cell Count and Reduces Odds of Anemia in a Mouse Model of Aging

**DOI:** 10.3390/antiox15060772

**Published:** 2026-06-22

**Authors:** Lindsey Lemus, Zachariah Agbala, Jessica Pettigrew, Steve C. Fordahl, Doreen Y. Larvie, Beatriz M. Fontoura, Nicholas B. Judd, Charelle S. Trim, Christopher D. Palmer, George L. Donati, Ian M. Carroll, Richard S. Bruno, Seth M. Armah

**Affiliations:** 1 Department of Nutrition, University of North Carolina Greensboro, Greensboro, NC 27412, USA; lgisenho@uncg.edu (L.L.); zlfisher@uncg.edu (Z.A.); jessica.pettigrew@duke.edu (J.P.); scfordah@uncg.edu (S.C.F.); doreen.larvie@ubc.ca (D.Y.L.); 2 Department of Chemistry, University of Scranton, Loyola Science Center, Scranton, PA 18510, USA; beatriz.fontoura@scranton.edu; 3 Department of Chemistry, Wake Forest University, Winston Salem, NC 27109, USA; nicholas.judd@agilent.com; 4 Laboratory of Inorganic and Nuclear Chemistry, Wadsworth Center, New York State Department of Health, Albany, NY 12237, USA; charelle.trim@health.ny.gov (C.S.T.); christopher.palmer@health.ny.gov (C.D.P.); george.donati@health.ny.gov (G.L.D.); 5 Department of Environmental Health Sciences, College of Integrated Health Sciences, State University of New York at Albany, Albany, NY 12208, USA; 6 Department of Nutrition, University of North Carolina Chapel Hill, Chapel Hill, NC 27599, USA; ian_carroll@med.unc.edu; 7 Human Nutrition Program, Department of Human Sciences, The Ohio State University, Columbus, OH 43210, USA; bruno.27@osu.edu

**Keywords:** anemia, aging, almonds

## Abstract

Anemia in aging is multifaceted and may be in part caused by increased inflammation and oxidative stress. Almonds contain nutrients that may improve inflammation or antioxidant activity, which can improve red blood cell (RBC) integrity and mitigate anemia. The aim of this study is to investigate the effects of almond consumption on hematologic markers in a mouse model of aging. Forty-eight 18-month-old male and female C57BL/6 mice were randomized to receive a control diet or an almond-supplemented diet (15% calories from almond meal) for 12 or 21 weeks (four groups, *n* = 12/group). Blood and tissues were collected after respective intervention periods for analyses of hematologic markers (complete blood count analysis) and antioxidant status (RBC alpha-tocopherol concentration and superoxide dismutase activity). Mice consuming the almond-supplemented diet had significantly lower red cell distribution width at 12 weeks (*p* = 0.05) and significantly higher RBC count at 21 weeks (*p* = 0.04). Regression analyses indicated that almond intake resulted in higher RBC count (β = 0.82, *p* = 0.026) and lower odds of anemia (OR = 0.20, *p* = 0.046) after adjusting for sex of mice and length of intervention. These findings suggest that almond intake in aging mice improved selected hematologic markers and reduced anemia risk.

## 1. Introduction

Anemia is a major public health concern, with almost one quarter of the world’s population suffering from some form of anemia [1]. Anemia may be caused by multiple factors, including poor dietary iron intakes and inflammation associated with aging or chronic diseases [2,3,4]. Deficiencies in nutrients such as vitamin B6, B12, vitamin E, and folate can also result in anemia [4,5]. Some forms of anemia (especially in the elderly) have also been classified as “unexplained” when decreased hemoglobin and erythropoietin levels are detected without an immediate known cause; unexplained anemia in the elderly may account for about one third of cases in people over 65 years of age [4,5,6]. Anemia from nutritional deficiencies may be addressed by oral supplementation or nutrition intervention; however, the other forms of anemia are often multifaceted and are therefore more complicated to address [2]. As the global population continues to age, addressing age-related anemia must be a public health priority due to its associations with mortality rates, cognitive decline, frailty, and other negative health outcomes in the elderly [2,6,7].

Anemia caused by inflammation is another major form of anemia that is observed in the aging population; inflammation induces anemia through different pathways: first, the upregulation of hepcidin (resulting in decreased iron absorption and increased iron sequestration) via increased interleukin-6 (IL-6) production [8,9]. Increased IL-6 triggers Janus kinase 2 (JAK2) to phosphorylate signal transducer and activator of transcription 3 (STAT3), translocating STAT3 to the nucleus and binding to its binding site in the proximal hepcidin promoter, upregulating hepcidin gene expression [3,7,8]. Inflammation also leads to an increase in reactive oxygen species released from macrophages, leading to the destruction or impairment of red blood cells when the red blood cell antioxidant system is overwhelmed [10]. Therefore, antioxidants that scavenge and/or detoxify reactive oxygen species (ROS) such as vitamin E, superoxide dismutase (SOD), catalase, and glutathione peroxidase are critical for the preservation of red blood cells and preventing anemia [8,10,11].

Nuts are recognized for their abundant health-promoting benefits. Almond intake, for instance, has been shown to lower the risk of cardiovascular disease, diabetes, and stroke by improving blood lipid profiles and lowering blood pressure parameters [12,13]. Almond consumption has also been shown to reduce inflammatory markers, including C-reactive protein (CRP) and IL-6 [14]. This may be attributed to its nutritional composition; a single serving of almonds contains approximately ~36% of the daily value (DV) for vitamin E, ~20% DV for magnesium, ~36% DV for manganese, 16% DV for copper, ~8% DV for zinc, and 13% DV for fiber [14,15]. In addition, almonds contain high concentrations of polyphenols (~312 mg/100 g of almond), which include hydrolysable tannins, proanthocyanidins, and flavonoids (as well as phenolic acids, lignans, isoflavones, and stilbenes to a lesser extent) and phytic acid or phytate (~0.35–9 g/100 g almond [15,16]. Our previous study and others have demonstrated that both phytate and polyphenols are associated with a reduction in inflammatory markers [14,17,18]. In addition, vitamin E in almonds is important for red blood cell (RBC) integrity due to its antioxidant properties, and the zinc and copper contents, which play important roles in the RBC antioxidant system as part of copper–zinc superoxide dismutase (Cu-Zn SOD) [19]. These properties of almonds make them an excellent candidate for reducing inflammation, improving the RBC antioxidant system, and mitigating oxidative damage of RBCs in aging, hence reducing the risk of anemia. However, no study to our knowledge has investigated the potential benefit of almond intake in reducing anemia risk in aging animal models. In this study, our aim is to investigate the effects of regular almond meal consumption on hematologic markers in aged C57BL/6 mice. We hypothesized that almond consumption would improve hematologic markers in an aging mouse model by increasing antioxidant activity and decreasing hepcidin concentrations. To evaluate this hypothesis, hematologic markers were compared between mice fed an almond-supplemented (AS) diet and mice fed a control diet for 12 or 21 weeks. Our findings are expected to advance our understanding of the roles of anti-inflammatory foods such as almonds in mitigating anemia.

## 2. Materials and Methods

### 2.1. Study Design and Dietary Intervention

**Figure 1:**
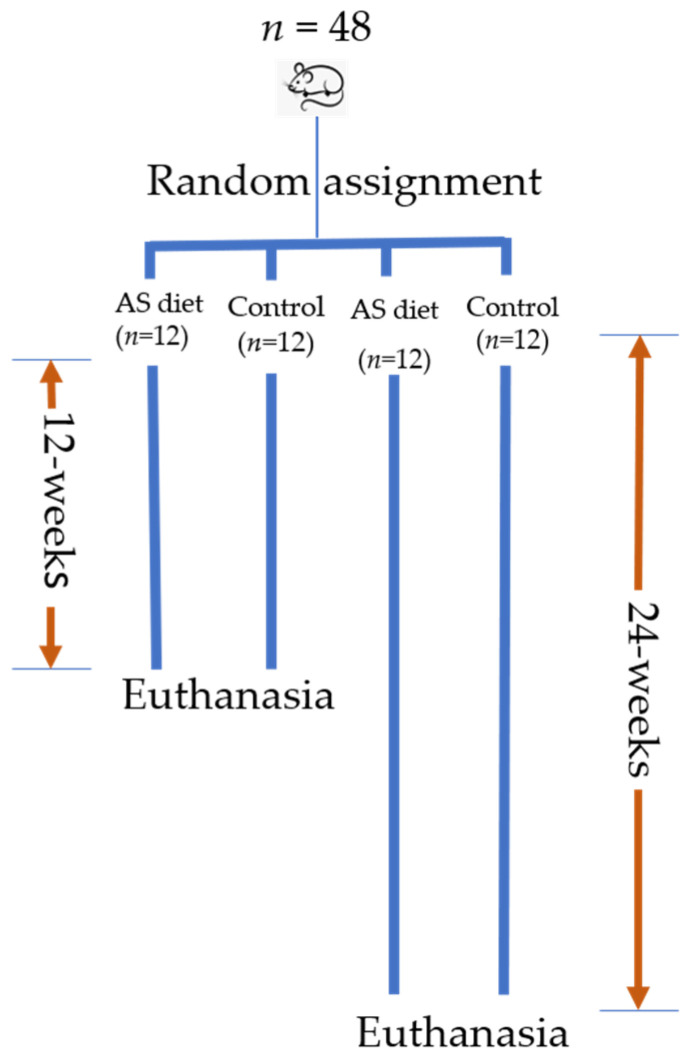
Study design. A total of forty-eight male and female C57BL/6 mice (*n* = 12 per group) were randomized to receive either a standard diet with 15% calories from fat (control diet) or the same diet supplemented with 15% calories from almond meal for 12- or 24-weeks. Tissue collection occurred after each respective time period. Due to observed increased mortality rate, the 24-week original design was amended to 21-weeks.

The animal study protocol was approved by the Institutional Animal Care and Use Committee of the University of North Carolina Greensboro (protocol #2022-1184). Forty-eight aging (18 months old) male and female C57BL/6 mice were randomized to receive either a control diet formulated with 15% calories from fat or the same diet supplemented with almond meal, contributing 15% of total calories (AS diet) for 12 weeks or 24 weeks (*n* = 12 per group, equal number of males and females). Study design is depicted in Figure 1. Due to an observed higher mortality rate among control mice, all mice in the 24-week cohort were euthanized after 21 weeks. Randomization was done manually by assigning each mouse an ID, writing the IDs on papers, mixing the folded papers in a container, and having a non-research staff member draw the mouse IDs into different groups. This was done separately for male and female mice to make sure there is an equal number of male and female mice in all groups. Research staff were only aware of the randomization results after all mice had been assigned to a group.

The sample size calculations were based on findings from our preliminary study, which indicated the number of mice needed to determine an effect size of 0.6 standard deviations (SD) in hemoglobin concentration as statistically significant with a power of 0.8 and a type I error rate of 0.05 to be *n* = 9 mice per group [20]. This was increased to *n* = 12 mice per group to consider age-related mortality that we anticipated in this study. The control and AS diets were energy-, total fat-, and iron-matched to address the confounding effects of dietary iron on hematologic markers. Finely ground almond meal (Trader Joe’s Grocery Store Company, Monrovia, CA, USA) was provided to Research Diets Inc. (New Brunswick, NJ, USA) for formulation of the AS diet. The control and AS diet compositions are shown in Table 1.

Mice were housed three mice per cage under standard pathogen-free conditions with free access to food and water. The room was kept at standard temperature (293–299 K), humidity ranged from 30 to 70%, and mice were kept under a 12:12 light–dark cycle. Paper for shredding and igloos for burrowing and nesting were provided to mice in each cage for enrichment activities. Mouse body mass was recorded once per week, and dietary intake was recorded three times per week until tissue collection. Humane endpoints for this study included infections unrelated to protocol, signs of moderate to severe pain that was not expected in the study plan, self-mutilation, abnormal weight loss exceeding 15% of free-feeding body weight relative to an age-matched reference, neurological disorders, cardiopulmonary disorders, abnormal feeding or defecation for 48 h that is unrelated to the study, non-weight bearing for more than 12 h, hypothermia, open wounds or ulcers, inability to ambulate and labored breathing that was not anticipated in the study plan.

### 2.2. Data Collection and Analysis

#### 2.2.1. Euthanasia, Blood, and Tissue Collection

At the end of each dietary intervention period, all mice were euthanized using isoflurane followed by rapid decapitation. Blood samples were collected from the trunk of the mice by jugular ejection into test tubes containing anticoagulant dipotassium ethylenediaminetetraacetic acid (K_2_EDTA) and inverted multiple times. Liver and spleen samples were collected and placed on ice, then stored at 193 K for future analysis.

#### 2.2.2. Blood Processing

The blood was centrifuged at 314.2 radian/second (rad/s) for 15 min at 277 K to separate plasma and red blood cells. The plasma was aliquoted into pre-labeled microcentrifuge test tubes and stored at 193 K. After aliquoting the plasma, the white buffy layer was removed and discarded, and the remaining red blood cells were subsequently divided into two tubes: one for vitamin E analysis and the other for antioxidant enzyme analysis.

#### 2.2.3. Analysis of Hematologic Markers

Complete blood count (CBC) analysis was performed using whole blood samples with the Abaxis VETSCAN ^®^ HM5 Hematology Analyzer from Zoetis Services (Zoetis Diagnostics LLC, Parsippany, NJ, USA). Immediately after euthanasia, approximately 50 µL of blood was collected in Microvette test tubes, inverted multiple times, and analyzed for CBC parameters (including RBC count, hematocrit (Hct), hemoglobin (Hgb), mean corpuscular volume (MCV), mean corpuscular hemoglobin (MCH), mean corpuscular hemoglobin concentration (MCHC), red cell distribution width (RDW), platelet count (PLT), and mean platelet volume (MPV)).

#### 2.2.4. RBC Antioxidant Enzyme Analysis

SOD activity in RBC lysate was determined using a commercially available enzyme-linked immunosorbent assay (ELISA) kit (Cayman Chemicals, Ann Arbor, MI, USA). The cells were first broken up by adding four parts of ice-cold high-performance liquid chromatography (HPLC)-grade water, resulting in a five-fold dilution of each sample. The resulting solution was centrifuged at 1047.2 rad/s at 277 K for 15 min, and the supernatant (erythrocyte lysate) was collected and stored at 193 K for future analysis. The ELISA manufacturer’s instructions were followed, and absorbance was read at 450 nanometer (nm) using a commercially available Epoch plate reader (Biotek Instruments Inc., Winooski, VT, USA).

RBC alpha-tocopherol concentrations were measured at the Bionutrition Core Laboratory at the Ohio State University (OSU). For alpha-tocopherol concentration measurement, RBCs were washed with phosphate-buffered saline (PBS) (pH 7.4) three times using equal parts of PBS and RBCs. The mixture was then centrifuged at 1047.2 rad/s at 277 K for 15 min, discarding the supernatant each time. After three washes, an equal volume of PBS containing 1.2% pyrogallol was added to the concentrated RBCs. Hgb was measured after processing using the Hemocue Hb201 system (Hemocue America, Brea, CA, USA), and the samples were stored at 193 K and later shipped on dry ice to OSU for RBC alpha-tocopherol analysis. Samples were saponified in ethanolic potassium hydroxide in ascorbic acid as described, with minor modifications [21]. Following extraction with hexane, samples were dried under nitrogen gas, reconstituted in methanol:ethanol (1:1), and injected on an Ultimate 3000 UPLC-ECD system (Thermo). Samples were then separated isocratically on a C18 column and detected at oxidation potentials of 150, 250, 350, and 450 mV. Quantification was performed at the dominant oxidation potential against peak areas of authentic standards traceable to NIST. RBC alpha-tocopherol for each sample was expressed per μg hemoglobin.

#### 2.2.5. Tissue and Plasma Analysis to Determine Trace Element Concentrations

Liver and spleen iron and zinc concentrations were measured in the Department of Chemistry at Wake Forest University by inductively coupled plasma optical emission spectrometry (ICP-OES) using a 5800 ICP-OES instrument from Agilent Technologies (Santa Clara, CA, USA). Copper concentrations in these samples were determined by inductively coupled plasma mass spectrometry (ICP-MS) using an 8800 ICP-MS/MS instrument from Agilent Technologies (Santa Clara, CA, USA). Samples were microwave-assisted digested with trace-metal-grade HNO_3_ and trace-analysis-grade hydrogen peroxide using an Ethos Up microwave-assisted digestion system from Milestone Inc. (Sorisole, BG, Italy). Iron and zinc were determined at the 238.204 and 213.807 nm emission wavelengths, while copper was determined at the mass-to-charge (*m*/*z*) of 63. To minimize spectral interferences, copper determination was carried out in single quadrupole mode using helium in the ICP-MS collision-reaction cell.

Plasma copper and zinc were measured in the Laboratory of Inorganic and Nuclear Chemistry, Wadsworth Center, New York State Department of Health, using an iCAP^TM^ TQ triple quadrupole inductively coupled plasma-mass spectrometer (ICP-MS/MS) (Thermo Scientific, Waltham, MA, USA). An aliquot of 50 µL of plasma sample was diluted with diluent solution to a total volume of 5 mL in a polypropylene autosampler tube (Labcon^®^ MetalFree^®^ Centrifuge Tubes, Petaluma, CA, USA) before analysis. The diluent solution comprised 0.5% *v*/*v* double-distilled nitric acid, 0.005% *v*/*v* Triton X-100^TM^, and 2 µg/L gallium (which was used as the internal standard to correct for any instrumental drift of any of the target analytes). Copper and zinc were, respectively, determined at *m*/*z* 65 and 66. Potential spectral interferences were minimized by employing oxygen gas (for iron and zinc) or helium gas (for copper) in the instrument’s collision-reaction cell (Q2). Zinc was measured in “high-high” resolution mode, i.e., the first quadrupole mass analyzer (Q1) was set at 1 amu and the third quadrupole (Q3) was set at 0.3 amu. Copper was measured using the instrument’s Kinetic Energy Discrimination (KED) mode, with Q3 set at a “high” 0.3 amu resolution. Three levels of internal human serum Quality Control (QC) material were analyzed at the start and end of each analytical run. The method accuracy was assessed by analyzing NIST Standard Reference Material (SRM) 1598 (Inorganic Constituents in Bovine Serum) and NIST 1598a (Inorganic Constituents in Animal Serum). Independent aliquots of the two SRMs were diluted in the same manner as the samples and placed on each analytical run.

#### 2.2.6. Plasma Hepcidin Analysis

Plasma hepcidin concentration was measured using a commercially available ELISA kit (LSBio, Newark, CA, USA). Assay manufacturer instructions were followed, and absorbance was read at 450 nm using a commercially available Epoch plate reader (Biotek Instruments Inc., Winooski, VT, USA).

### 2.3. Statistical Analysis

Data were analyzed using R software (Version 4.3.3) [22]. All mice with data were included in each analysis. Means and standard errors were reported for outcome variables by intervention groups. Independent t-tests were used to compare hematologic markers between the control and AS groups at 12 weeks and 21 weeks. Paired t-tests were used for pre-and post-body weight comparison in each diet group. The primary outcome for this study was hemoglobin concentration. Secondary outcomes include other hematologic markers (RBC count, Hct, MCH, MCHC, and RDW), plasma hepcidin concentration, and antioxidant activity (RBC SOD activity and RBC alpha-tocopherol concentration). Linear regression analysis was used to determine the relationship between almond consumption and hematologic and inflammatory markers, adjusting for sex and length of intervention. Beta coefficients and their 95% confidence intervals (CIs) were reported for linear regression analysis. Logistic regression was used to determine the relationship between almond consumption and risk of anemia, adjusting for sex and length of intervention. Odds ratios (ORs) and their 95% CIs were reported for logistic regression. Mediation analysis was conducted to determine the mediation effect of several factors on the relationship between almond intake and anemia risk in aged mice. Statistical significance was set at *p* ≤ 0.05.

## 3. Results

### 3.1. Survival Rates, Food Intake and Body Weight

Among the aged mice in the 12-week cohort, three mice (25%) died prior to scheduled euthanasia in the control group compared to one mouse (8%) in the AS group. In the 21-week cohort, four mice (33%) died in the control group by week 21 compared to two mice (17%) in the AS group, prompting us to terminate the study to have sufficient mice to meet the sample size per group expectation (*n* = 9 mice per group in each cohort). Thus, a total of seven mice died or were euthanized earlier in the control group compared to a total of three in the AS group throughout the study.

Mean daily food and iron intakes for all mice were 3.2 ± 0.07 g/d and 0.16 ± 0.00 mg/d, respectively. Food intake did not differ significantly between the control and AS groups for both 12 (3.2 ± 0.1 g/d vs. 3.4 ± 0.1 g/d) and 21-week (3.3 ± 0.1 g/d vs. 3.1 ± 0.2 g/d) cohorts (*p* > 0.05 for both). Similarly, mean daily iron intake was not significantly different between control and AS groups for 12 (0.16 ± 0.01 mg/d vs. 0.15 ± 0.01 mg/d) or 21-week (0.17 ± 0.01 mg/d vs. 0.15 ± 0.01 mg/d) cohorts (*p* > 0.05 for both). Among mice receiving the control or AS diets for 12 weeks, mean body weight did not differ significantly between groups at both baseline and 12 weeks (39.0 ± 1.5 g for control vs. 38.8 ± 2.5 g for AS, *p* = 0.95 at baseline and 35.4 ± 1.1 g for control vs. 35.6 ± 2.2 g for AS, *p* = 0.94 at 12 weeks). While no significant differences were observed between groups for baseline and end-of-treatment weight, both demonstrate significant weight loss from their respective baseline measurements (*p* ≤ 0.05 for both groups).

Similarly, among mice receiving the control or AS diets for 21 weeks, no significant difference was observed at either baseline or 21 weeks (39.1 ± 1.6 for control vs. 42.0 ± 1.6 g for AS, *p* = 0.22 at baseline and 39.5 ± 1.3 for control vs. 41.2 ± 1.3 g for AS, *p* = 0.48 at 21 weeks). Also, there were no significant within-group changes in weight from baseline to the end of the intervention (*p* > 0.05 for both groups).

### 3.2. Hematologic Markers

Differences in hematologic markers by treatment and length of intervention are shown in Table 2 for aged mice. There were no significant differences in Hgb concentrations between groups at 12 or 21 weeks (*p* > 0.05). RDWsd, which reflects the variation in size of RBCs, was significantly lower in the AS group compared to the control group at 12 weeks (32.3 ± 1.1 fl for AS vs. 37.8 ± 2.8 fl for control, *p* = 0.05), while no significant difference was seen at 21 weeks. RBC count was significantly higher in the AS group compared to the control group at 21 weeks (11.2 × 10^12^ ± 0.3 for AS vs. 10.4 × 10^12^ ± 0.4 for control, *p* = 0.04), while no significant between-treatment difference was observed at 12 weeks. There were no significant differences in other hematologic markers between treatment groups at 12 or 21 weeks.

### 3.3. Antioxidant Activity, Hepcidin, and Trace Element Concentrations

RBC SOD activity was higher in the AS groups compared to the control groups at 12 weeks; however, the difference only trended towards significance at 12 weeks (102.4 ± 17.0 U/mL for AS vs. 70.3 ± 13.3 U/mL for control, *p* = 0.08) and was not significant at 21 weeks. There were no significant differences in alpha-tocopherol, plasma hepcidin, and liver and spleen iron concentrations between diet groups at 12 or 21 weeks.

### 3.4. Odds of Anemia and Mediation Effect

Mice were classified as anemic if Hgb concentration was less than 12 g/dL [23]. Among all mice with hemoglobin data (*n* = 44), 11 (25%) developed anemia, including mice from all treatment groups. When broken down by groups, three mice (13%) in the AS group developed anemia (one in the 12-week cohort and two in the 21-week cohort) compared to eight mice (38%) in the control group (five in the 12-week cohort and three in the 21-week cohort). Logistic regression analysis showed that the odds of anemia were significantly lower in the AS group compared to the control group (OR (95%CI): 0.20 (0.03, 0.88): *p* = 0.046 (Table 3). This effect of almonds was not significant once we adjusted for SOD activity. Therefore, we conducted a mediation analysis to determine if SOD mediated the effect of almonds on anemia risk (Table 4). RBC SOD activity had a weak partial mediation effect (average causal mediation effect (ACME (95% CI ) of –0.10 (−0.25, 0.01); *p* = 0.074) with a significant total effect (Total effect (95% CI): −0.24 (−0.49, 0.00), *p* = 0.047). We also assessed whether RBC count mediated the effect of almond intake on anemia risk. RBC count had a statistically significant ACME (95% CI): −0.18 (−0.39, −0.01), *p* = 0.026) with a non-significant average direct effect (ADE) of −0.07 (−0.3, 0.19) (*p* = 0.52). Finally, we tested whether SOD mediated the effect of almond intake on RBC count, and found no significant causal mediation (*p* = 0.19) but rather a weak direct effect of almond intake on RBC count (ADE (95% CI): 0.66 (−0.04, 1.38); *p* = 0.070) with a significant total effect (total effect (95% CI): 0.81 (0.10, 1.52); *p* = 0.021).

## 4. Discussion

The purpose of this study was to investigate the effects of almond intake on hematologic markers in aging mice. After 12 and 21 weeks of dietary intervention, hemoglobin concentration did not differ significantly between the AS and control groups, and regression analysis showed no significant associations between almond intake and hemoglobin concentration after adjusting for sex and length of intervention. However, when mice were classified as anemic or non-anemic, we observed significant differences in anemia prevalence by diet group, specifically among the 12-week cohort, with lower anemia prevalence in the AS group compared to the control group (*p* = 0.038). Our findings convey two important points: first, the high prevalence of anemia in the aging mice in this study (25%) and particularly in the control group (38%) suggests that aging C57BL/6 mice represent a non-invasive model for age-induced anemia. For many years, mice have been a reliable model for studying the aging process due to their short life spans and genetic similarity to humans, and researchers have relied on the aging mouse model to exemplify a “healthy” aging process [24]. Based on our findings related to the prevalence of anemia in non-disease-induced aged mice, we see that biological age may be independently linked to a decline in hemoglobin and hematologic health. Furthermore, many preclinical in vivo models of anemia are narrowly focused on anemia of inflammation and are invasive, requiring induction of a state of inflammation using various inflammatory molecules such as lipopolysaccharide, turpentine, or peptidoglycan-polysaccharide, or injection of heat-killed bacteria such as *Brucella abortus* [25,26,27,28,29,30,31,32,33]. These substances are commonly injected intraperitoneally, and mice are either subjected to this procedure periodically over a short amount of time (4–6 weeks) or are euthanized shortly after exposure to inflammatory molecules to determine the effects of inflammation on hematologic markers [25,26,27,28,29,31]. These models may be appropriate for certain studies; however, our study has demonstrated that C57BL/6 mice are a suitable, less invasive model for studies focused on the decline in functional iron status during aging, similar to what is seen in humans [4]. Our results also corroborate findings from a previous study that showed aging C57BL/6 mice represent an appropriate mammalian model of anemia in aging [34].

Second, the lower anemia rate in the AS group compared to the control group suggests a beneficial effect of almond supplementation on anemia risk in an aging mouse model. In further analysis, we used logistic regression adjusted for the length of intervention and sex to investigate this relationship. We found that the odds of anemia were significantly lower in mice consuming the AS diet compared to the control group, further supporting the assertion of the beneficial effect of almonds. Our original hypothesis was that almond supplementation would reduce anemia risk by reducing hepcidin concentration and improving RBC antioxidant activity. This is based on several anti-inflammatory and antioxidant nutrients/non-nutrients such as vitamin E, zinc, polyphenols, and phytates found in almonds [35,36]. However, we observed no significant difference in hepcidin concentration between the AS and control mice, indicating that the hepcidin-ferroportin axis was not a major contributing factor to the observed anemia. When we examined the role of antioxidant activity in iron status, we observed that mice fed a diet supplemented with almonds for 12 weeks had higher SOD activity compared to mice fed the control diet, with the difference trending towards statistical significance (*p* = 0.08). We also observed that when SOD activity was accounted for in the logistic regression model that predicted anemia risk, almond intake was no longer associated with reduced odds of anemia, suggesting that improved SOD activity may be contributing to the lower odds of anemia observed in the aging mice. Previous research has shown that mice supplemented with almond polysaccharides had increased SOD activity and improvements in dextrose sodium sulfate (DSS)-induced colonic tissue damage [37]. In male rats treated with scopolamine to induce oxidative stress and impaired memory, almond intake was shown to significantly increase SOD, glutathione peroxidase, and catalase levels compared to controls [38]. A recent meta-analysis has also shown that almond supplementation significantly increased SOD activity in a variety of human populations [39]. In contrast, we found no significant differences in alpha-tocopherol concentrations between diet groups.

We observed that RBC count was significantly higher in mice fed a diet supplemented with almonds compared to mice fed a control diet at 21 weeks. Our mediation analysis indicated that the increase in RBC count was a significant factor in reducing anemia risk. Altogether, these results indicate that RBC count fully mediated the relationship between almond supplementation and odds of anemia. This provides evidence that the positive effect of almonds on anemia may be related to their impact on RBC production or preservation. Erythropoiesis is crucial for iron homeostasis and is impaired during the aging process as hematopoietic stem cells (HSC) become senescent. A decline in erythropoiesis implies decreased RBC count, and in turn decreased hemoglobin levels that are often seen in the elderly [4,40]. Our study also showed that mice fed a diet supplemented with almonds had significantly decreased red cell distribution width at 12 weeks. While a routine parameter collected in a CBC analysis, RDW can be an early indicator of iron dysregulation. RDW has been reported to be positively associated with proinflammatory cytokines, including CRP and IL-6, and as an independent predictor of mortality in community-dwelling older adults irrespective of the presence of an age-related disease [41]. Future studies on the implications of the effects of almonds on RDW are recommended.

Our study has several strengths. First, the amount of almond meal used in this study (15% of total calories) corresponds to an amount appropriate to include in a typical human diet. Secondly, we were able to demonstrate through mediation analysis how almond supplementation reduces the odds of anemia in an aging mouse model. On the other hand, our study would have benefited from including several time points for the measurement of biomarkers to understand the trends in the relationship between almond intake and iron status. Furthermore, the small sample sizes combined with poor survival rates in aging mice may have impacted secondary outcomes such as antioxidant activity, hepcidin, and hematologic markers. Finally, despite the importance of our findings, these results cannot be extrapolated to humans, implying the need for a human study to further investigate the relationship between almond intake and hematologic markers.

## 5. Conclusions

Overall, the findings of our study demonstrate that an almond meal-rich diet reduces the risk of anemia in an aging mouse model through increased RBC count. These findings contribute significantly to anemia research by demonstrating an alternate approach to reducing anemia risk outside traditional approaches such as nutrient supplementation or consumption of iron-rich foods.

## Figures and Tables

**Table 1:** Nutritional composition (g/100 g) of control and almond-supplemented (AS) diets.

Nutrient/Ingredient	Control Diet	AS Diet
Protein	17.2	17.2
Carbohydrate	64.8	64.7
Sugars	7.7	8.1
Fiber	4.9	4.8
Fat	6.4	6.4
Saturated fat	1.5	0.8
Monounsaturated fat	1.8	3.5
Polyunsaturated fat	2.8	1.8
Trans fat	0.01	0.00
Iron	0.005	0.005
Almond meal	0	10

**Table 2:** Hematologic markers and tissue trace mineral concentrations in mice fed a control diet or an almond-supplemented (AS) diet for 12 or 21 weeks.

	12 Weeks					21 Weeks				
	AS (*n* = 11)		Control (*n* = 10)		*p*-Value	AS (*n* = 12)		Control (*n* = 11)		*p*-Value
	Mean	SE	Mean	SE		Mean	SE	Mean	SE	
Hemoglobin ^1^ (g/dL)	12.7	0.4	12.1	0.6	0.21	13.5	0.4	12.7	0.5	0.12
Anemia prevalence (%)	9	9	50	16	0.04 *	17	11	27	13	0.54
Red blood cell count (×10^12^)	10.5	0.4	9.8	0.4	0.14	11.2	0.3	10.4	0.4	0.04 *
Hematocrit (%)	45.2	1.4	43.7	1.4	0.23	47.4	1.1	44.8	1.4	0.08
Mean corpuscular volume (fL)	43.4	1.1	44.7	1.1	0.80	42.4	0.8	43.4	1.1	0.75
Mean corpuscular hemoglobin (pg)	12.2	0.3	12.3	0.3	0.49	12.0	0.2	12.3	0.3	0.79
Mean corpuscular hemoglobin concentration (g/dL)	28.1	0.2	27.5	0.7	0.20	28.3	0.4	28.4	0.5	0.53
Red cell distribution width, cv ^2^ (%)	21.5	0.3	22.8	1.1	0.12	21.3	0.3	21.9	0.8	0.22
Red cell distribution width, sd ^3^ (fl)	32.3	1.1	37.8	2.8	0.05 *	31.1	0.7	33.0	1.5	0.14
Platelet count (×10^9^)	647.4	33.4	765.5	27.8	0.01 *	559.6	66.0	613.0	81.5	0.31
Plasma hepcidin (ng/mL)	14.3	1.8	15.0	2.5	0.42	13.9	2.7	12.4	1.7	0.68
Superoxide dismutase activity (U/mL)	102.4	17.0	70.3	13.3	0.08	79.2	5.6	69.4	9.2	0.19
RBC α-Tocopherol (µmol/µg Hgb)	28.48	3.52	38.35	12.01	0.78	26.57	5.24	35.58	9.52	0.79
Plasma copper (µg/dL)	81.9	6.3	96.3	10.9	0.86	94.1	12.0	101.0	10.9	0.66
Plasma zinc (µg/dL)	65.0	5.3	69.3	5.1	0.72	76.2	4.6	89.4	9.2	0.89
Liver iron (µg/g)	231.1	22.7	200.3	22.7	0.18	172.4	22.2	148.8	20.3	0.22
Liver copper (µg/g)	4.5	0.3	5.0	0.2	0.92	3.5	0.2	4.0	0.3	0.92
Liver zinc (µg/g)	28.1	1.7	29.0	3.6	0.59	21.8	1.7	26.0	3.5	0.85
Spleen iron (µg/g)	979.3	243.0	651.6	134.9	0.13	1158.2	290.2	1017.2	197.0	0.35
Spleen copper (µg/g)	1.1	0.08	1.2	0.09	0.82	1.0	0.08	1.0	0.07	0.58
Spleen zinc (µg/g)	14.4	1.5	12.5	1.6	0.21	12.3	2.0	11.6	1.7	0.40

* *p* ≤ 0.05, ^1^ hemoglobin was measured using the HM5 Vetscan (Zoetis Diagnostics LLC, Parsippany, NJ, USA); ^2^ cv: coefficient of variation; ^3^ sd, standard deviation.

**Table 3:** Relationship between almond supplementation and selected iron status biomarkers in aging mice adjusted for sex and length of intervention.

	Hemoglobin, g/dL (*n* = 44)		Hematocrit, % (*n* = 44)		RBC Count, ×10^12^ (*n* = 44)		Liver Iron, µg/g (*n* = 43)		Spleen Iron, µg/g (*n* = 44)		Anemia (*n* = 44)	
	β	SE	β	SE	β	SE	β	SE	β	SE	OR	95% CI
Treatment ^1^												
Almond supplementation	0.73	0.46	1.99	1.34	0.82 *	0.35	23.1	20.3	151	190	0.2 *	0.03, 0.88
Sex ^2^												
Male	0.48	0.46	−1.01	1.34	0.70	0.35	−54.5 *	20.3	−845 *	191	0.32	0.06, 1.4
Length of intervention ^3^												
24 weeks	0.06	0.04	0.14	0.11	0.05	0.03	−4.7 *	1.7	22	16	0.64	0.14, 2.8

* *p* ≤ 0.05, ^1^ reference group (treatment): control diet, ^2^ reference group (sex): female, ^3^ reference group (length of intervention): 12 weeks.

**Table 4:** Mediation analysis on the effects of almond intake on RBC count and anemia risk in aging mice (*n* = 44).

Mediator	Outcome		Estimate	95% CI Lower	95% CI Upper	*p*-Value
RBC SOD activity	Anemia	ACME	−0.1008	−0.2465	0.01	0.074
		ADE	−0.1424	−0.3781	0.07	0.179
		Total Effect	−0.2432	−0.4871	0	0.047 *
		Proportion Mediated	0.4008	−0.226	1.64	0.105
RBC SOD activity	RBC count	ACME	0.1559	−0.0604	0.5	0.192
		ADE	0.6588	−0.0435	1.38	0.070
		Total Effect	0.8147	0.1031	1.52	0.021 *
		Proportion Mediated	0.1685	−0.132	1	0.208
RBC Count	Anemia	ACME	−0.1788	−0.3869	−0.01	0.026 *
		ADE	−0.0693	−0.3013	0.19	0.515
		Total Effect	−0.248	−0.5215	0.06	0.089
		Proportion Mediated	0.6829	−0.8459	3.17	0.097

* *p* ≤ 0.05. ACME, Average causal mediation effect; ADE, Average direct effect; RBC, Red blood cell; RBC SOD, Red blood cell superoxide dismutase.

## Data Availability

The datasets presented in this article are not readily available because the data are part of an ongoing study. Requests to access the datasets should be directed to Seth Armah (s_armah@uncg.edu).

## Abbreviations

The following abbreviations are used in this manuscript:

JAK2	Janus kinase 2
STAT3	Signal transducer and activator of transcription 3
ROS	Reactive oxygen species
SOD	Superoxide dismutase
CRP	C-reactive protein
DV	Daily value
IL-6	Interleukin-6
RBC	Red blood cell
Cu-Zn SOD	Copper–Zinc-dependent superoxide dismutase
AS diet	Almond-supplemented diet
K_2_EDTA	Dipotassium ethylenediaminetetraacetic acid
Rad/s	Radian/second
CBC	Complete blood count
Hct	Hematocrit
Hgb	Hemoglobin
MCV	Mean corpuscular volume
MCH	Mean corpuscular hemoglobin
MCHC	Mean corpuscular hemoglobin concentration
RDW	Red cell distribution width
PLT	Platelet count
MPV	Mean platelet volume
HPLC	High-performance liquid chromatography
ELISA	Enzyme-linked immunosorbent assay
nm	nanometers
PBS	Phosphate-buffered saline
ICP-OES	Inductively coupled plasma optical emission spectrometry
ICP-MS	Inductively coupled plasma mass spectrometry
KED	Kinetic Energy Discrimination
QC	Quality Control
SRM	Standard reference material
CIs	Confidence intervals
OR	Odds ratio
SE	Standard error
ACME	Average causal mediation effect
ADE	Average direct effect
DSS	Dextrose sodium sulfate
HSC	Hematopoietic stem cell
NIDDK	National Institute of Diabetes and Digestive and Kidney Diseases
NIH	National Institute of Health
NSF-MRI	National Science Foundation’s Major Research Instrumentation Program
